# Vote-processing rules for combining control recommendations from multiple models

**DOI:** 10.1098/rsta.2021.0314

**Published:** 2022-10-03

**Authors:** William J. M. Probert, Sam Nicol, Matthew J. Ferrari, Shou-Li Li, Katriona Shea, Michael J. Tildesley, Michael C. Runge

**Affiliations:** ^1^ Big Data Institute, Li Ka Shing Centre for Health Information and Discovery, Nuffield Department of Medicine, University of Oxford, Oxford, UK; ^2^ CSIRO Land and Water, 41 Boggo Road, Dutton Park, Queensland, Australia; ^3^ Center for Infectious Disease Dynamics, Department of Biology, Eberly College of Science, The Pennsylvania State University, University Park, PA, USA; ^4^ Department of Biology and Intercollege Graduate Degree Program in Ecology, 208 Mueller Laboratory, The Pennsylvania State University, University Park, PA, USA; ^5^ State Key Laboratory of Grassland Agro-ecosystems, Center for Grassland Microbiome, and College of Pastoral, Agriculture Science and Technology, Lanzhou University, Lanzhou, People's Republic of China; ^6^ Zeeman Institute for Systems Biology and Infectious Disease Epidemiology Research, School of Life Sciences and Mathematics Institute, University of Warwick, Coventry, CV4 7AL, UK; ^7^ US Geological Survey, Eastern Ecological Science Center at the Patuxent Research Refuge, 12100 Beech Forest Road, Laurel, MD, USA

**Keywords:** epidemiology, voting, management, decision-making, multiple models

## Abstract

Mathematical modelling is used during disease outbreaks to compare control interventions. Using multiple models, the best method to combine model recommendations is unclear. Existing methods weight model projections, then rank control interventions using the combined projections, presuming model outputs are directly comparable. However, the way each model represents the epidemiological system will vary. We apply electoral vote-processing rules to combine model-generated rankings of interventions. Combining rankings of interventions, instead of combining model projections, avoids assuming that projections are comparable as all comparisons of projections are made within each model. We investigate four rules: First-past-the-post, Alternative Vote (AV), Coombs Method and Borda Count. We investigate rule sensitivity by including models that favour only one action or including those that rank interventions randomly. We investigate two case studies: the 2014 Ebola outbreak in West Africa (37 compartmental models) and a hypothetical foot-and-mouth disease outbreak in UK (four individual-based models). The Coombs Method was least susceptible to adding models that favoured a single action, Borda Count and AV were most susceptible to adding models that ranked interventions randomly. Each rule chose the same intervention as when ranking interventions by mean projections, suggesting that combining rankings provides similar recommendations with fewer assumptions about model comparability.

This article is part of the theme issue ‘Technical challenges of modelling real-life epidemics and examples of overcoming these’.

## Highlights

— When multiple models inform decisions it is not clear how to combine recommendations.
— Electoral vote-processing rules can combine ranks of interventions from model projections.
— Combining ranks avoids assuming different model projections are directly comparable.
— In two outbreak examples, each rule chose the same interventions as when ranking mean projection.
— The robustness of ranking interventions was affected by adding biased models.
— Adding additional noisy models had little impact on choice of intervention.

## 1. Introduction

Decision-making during disease outbreaks is often assisted by mathematical models that can either generate forecasts of disease dynamics or compare the impact of control interventions. Mathematical models of disease outbreaks can take many forms, and consulting an array of models may mean sensitivity analyses across model assumptions naturally run in parallel (i.e. by different modelling teams; e.g. [1]), important when time is short, and can lead to improved forecast accuracy (e.g. [2]).

An abundance of models is present in the context of both human and animal health: Kao [3] outlines at least four models used during the 2001 foot-and-mouth disease (FMD) outbreak in the UK; Pomeroy *et al*. [4] cite 17 articles that use mathematical models of control based on the FMD 2001 outbreak in the United Kingdom (UK); Eaton *et al*. [5] used 10 models to forecast human immunodeficiency virus (HIV) incidence, prevalence and antiretroviral coverage for the HIV epidemic in South Africa; Li *et al*. [6] identified 37 mathematical models for Ebola transmission in West Africa and provided projections of control interventions; and Shea *et al*. [1] elicited projections from 16 modelling teams to inform county reopening strategies in the United States (US) in the midst of the COVID-19 pandemic.

Many national and international consortia capitalize on the abundance of models by eliciting projections from multiple modelling groups to help guide decision-making in real time. This has been carried out in a number of contexts: the COVID-19 Scenario Modeling Hub [7], the COVID-19 Forecast Hub [8], Scientific Pandemic Influenza Group on Modelling, Operational sub-group (SPI-M-O) for the Scientific Advisory Group for Emergencies (SAGE) in the UK [9], the Vaccine Impact Modelling Consortium [10] and the Intergovernmental Panel on Climate Change (e.g. [11]). A plurality of models can lead to challenges within the decision-making process: concordance in model output may corroborate policy recommendations but where model outputs are discordant it can be difficult to determine the best control intervention.

Models are often developed through a process of fitting to retrospective data; having done this the suite of models may then be used to project future scenarios. There is a large and robust literature about the former step; though in many cases (e.g. a novel (re)emerging outbreak) such retrospective data do not yet exist and models are still useful in scenario analysis. In this analysis, we focus on methods for combining the results of scenario projections to inform decision-making. The consortia mentioned above synthesize model projections in a number of ways to inform decision-making, typically using some form of model averaging (see below). We posit that in the context of decision-making, where the focus is on using models to compare different interventions, it may be preferable to first rank the interventions within each model's results and then combine rankings across different models to determine the best course of action. We suggest this can be done with vote-processing rules, as are commonly used in electoral voting systems. We first provide background on different approaches to synthesizing model projections, investigate why this can cause issues in decision-making, and then outline vote-processing rules in more detail.

In response to the plurality of mathematical models, ‘ensemble modelling’ methods have been developed that synthesize output from multiple models. The idea that combining the results of multiple models can yield better predictions than individual models (provided the component models contain independent information) has been around for more than 50 years [12]. Ensemble modelling may seek either a consensus (usually via some form of model weighting) or to generate probabilistic ‘bounding boxes’ that represent uncertainty among the model predictions [13]. While there is great value in representing uncertainty, decision-makers need to select a preferred course of action, so we restrict the scope of our manuscript to methods for selecting a consensus intervention. Consensus ensemble modelling combines distributions of predictions from different models via a weighting scheme (hereafter ‘model averaging’). Ensemble modelling approaches have been used in weather prediction and meteorology [14,15], to predict species occurrence ecology [13,16], in fisheries [17], in epidemiology for FMD control [18,19] and COVID-19 indicators and management [1,7,9]. While model ensembles have been shown to outperform individual models, debate remains about the best way to combine and weight models [13].

In any multi-model project, questions arise over which models to include. We briefly address three such concerns since addressing these concerns provides justification for investigating approaches to aggregating output from models. We address the following three concerns: the use of models beyond the ‘best-fitting’ model, the absolute number of models to include and whether any criterion should be applied to include or exclude models. Firstly, although data fitting is a major part of modelling, and each individual modelling team needs to be concerned with model fitting, typically the independent consortia that run multi-model exercises (mentioned above) will be concerned with aggregation of model projections and will not focus on model calibration and fitting (although they will still compare projections with data). Regardless of how well models fit data and how they are calibrated, the question still remains as to how to combine model output—the central question addressed in this work. Second and thirdly, we believe the question of how many (and which) models to include in a multi-model project is an open question. There is also not a clear consensus in the literature as to the number of models and complexity of models to include, as this may depend on the objective of the multi-model project. Some research calls for carefully chosen and curated models (e.g. [20]), while others suggest open calls are best given (e.g. [21]). We cannot find justification in the literature for using a curation process but we provide evidence against using a curation process. Should a curation process of which models to include be carried out then it is not clear who is to decide which model is ‘appropriate’ to include and we do not believe there is published evidence that the results from a curated group of models would have better performance than using an open call. Without being transparent regarding inclusion criteria, excluding models would introduce additional biases that will be based on the subjectivity of the researcher(s) deciding on which models to include. To evidence this, for instance, we highlight that the COVID-19 pandemic saw several models were initially repurposed from influenza modelling (deemed appropriate by subject experts), yet SARS-CoV-2 was later shown to have very different dynamics to influenza (e.g. in regard to children and the importance of asymptomatic/pre-symptomatic transmission). Furthermore, there are studies in the expert elicitation literature that experts do not provide better predictions than non-experts (e.g. [22]) and that using a diversity of experts leads to better calibrated results. We believe this provides some evidence for such a phenomenon to exist in modelling too and therefore supports using an open call to increase the diversity of models and modelling teams.

Combining model output using model averaging raises important questions about the recommended course of action. First, because the results are averaged, the recommended course of action does not represent any ‘real’ model, so it is difficult to relate the output to the model inputs. While individual models may lack the diversity of assumptions of an averaged model set, averaging may miss the opportunity to highlight model assumptions that may lead to differences in recommendations. Essentially, the average model represents a new situation that may not be at all representative of any of its component parts, and the severity of this is impossible to judge until after the recommended action has been implemented. Second, the candidate models may provide an output that has different units (due to different model assumptions) such that the appropriate method to average results is unclear (this can be present even if model outputs are ostensibly the same—e.g. the example on ‘hospitalized cases’ below). Third, model averaging is sensitive to outliers which can bias the results away from median performance.

Consider the following simplified example that illustrates some of these issues (table 1). Our example consists of four models that are selecting between two actions. For the purposes of the example, we aim to maximize the model outcome. We assume that all four models apply the following simple decision rule: if the model outcome is less than 25, then select action 1; else select action 2. All models are assumed to have equal support and therefore are given equal weight in the average. Three of the four models recommend selecting action 1, yet the extreme prediction of model 2 biases the model set and forces the average score to select action 2. This seems at odds with the outcomes, because the averaged output recommends supporting the action recommended by the outlier rather than the action supported by the majority of the models. Looking at the discordance in the model results, we might expect that models 1, 3 and 4 are all modelling a similar process and that model 2 is modelling a different process. Assuming that we believe one of the modelled processes best describes the system, it seems desirable that the averaged output should be similar to one of the two extremes. However, the averaged outcome sits between the extremes, having no obvious relation to either the three models that agree (range 0–20), nor the extreme estimate of 200; this seems inconsistent with the inputs and begs the question of what the averaged output means.

**Table 1.** A simple example decision-making problem.

	Model 1	Model 2	Model 3	Model 4	average (mean)
predicted outcome	0	200	10	20	57.5
recommended action	action 1	action 2	action 1	action 1	action 2

The example suggests that selecting actions from model averaging is vulnerable to the addition of models that make extreme or discordant predictions.

When using epidemiological models to guide decision-making, the key question for decision-makers is rarely ‘which model is correct?’, but instead ‘what is the best course of action?’. This is an important distinction. For example, Li *et al*. [6] showed, in the context of Ebola control, that the absolute projections from models can vary wildly but estimates of the relative efficacy of different control strategies can be highly concordant. Similarly, in the context of FMD control, Probert *et al*. [23] illustrated that as an outbreak progresses, and we learn about the system under study, the relative efficacy of control interventions can be resolved reasonably early in an outbreak despite large scale differences in the absolute magnitude of projections at the same point in time.

**Figure 1.**
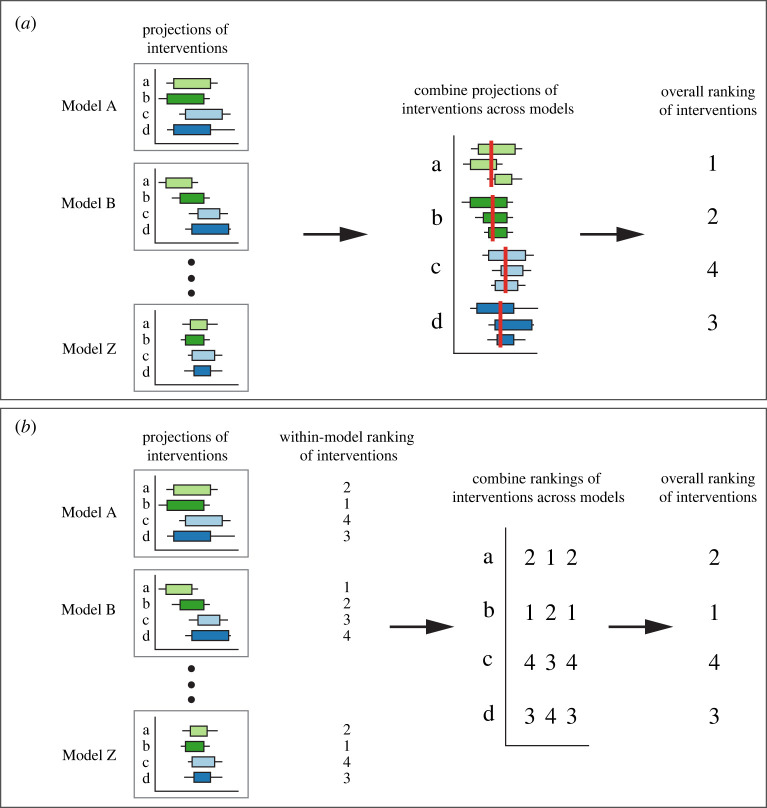
Two paradigms for combining model outputs to compare control interventions: (*a*) combining projections of the efficacy of control interventions across all models and ranking the ensemble predictions, and (*b*) combining rankings of control interventions, generated within each model, using vote-processing rules (the approach outlined in this work; the figure using the sum of ranks to make a final rank). In all graphs, the horizontal axis is meant to represent a metric such as the size of the outbreak, which the decision-maker would like to minimize. (Online version in colour.)

How does one generate recommendations for the best course of action from multiple models? A useful approach is to conduct sensitivity analyses: if all models suggest the same course of action across different parameterizations then the decision is clear and there is no need for combining models [6,24,25], but if models provide conflicting recommendations as to the best intervention, another approach is required. Meteorological forecasts are rarely (if ever) used to simulate control interventions but the ensemble prediction, using all combined model results, can be used in ranking the impact of different control interventions (figure 1*a*).

Another approach is to first rank interventions within each model and then synthesize rankings of interventions across all models. Combining rankings of control interventions that are generated from each model, therefore, provides an ‘ensemble of rankings' (figure 1*b*). By combining ordinal rankings of interventions across models, instead of the projections, we avoid making assumptions regarding how units of the projections from each model relate to one another (i.e. the comparability of model projections).

For example, if two independently developed epidemiological models make projections of ‘hospitalized cases’ of a disease, it may seem sound to combine projections from these models on that metric for the same intervention. However, the term ‘hospitalized cases’ is non-specific and may reflect a range of underlying assumptions by modelling groups; the common term may provide a false sense of security as to how similar this outcome is in these two models (so-called ‘linguistic imprecision’ or ‘linguistic uncertainty’; [21,26]). Combining ‘hospitalized cases’ from these two models requires implicitly deciding how numbers from one model correspond to numbers from another model. If the aim is to compare interventions, then relying on within-model comparisons of ‘hospitalized cases’ across interventions will keep any unforeseen assumptions constant across such projections. Harmonizing model outputs will only become more challenging as models increase in complexity and variability in complexity of contributing models increases [1], for instance, including both compartmental and individual-based models.

Electoral voting systems, as used in the election of government officials, typically generate a single winner, or a ranked list, from ordinal data of voters' preferences (i.e. votes). Such methods for combining rankings have been evaluated under various criteria, including the ability to represent voters' preferences, susceptibility to tactical voting (or ‘strategic voting’; whereby a voter may not vote for their first preference to avoid a particular outcome; [27]), ease of understanding, and ease of use/computation (see references under each vote-processing rule in Methods).

In this paper, we apply four vote-processing rules, as used in electoral voting systems, to process the rankings of control interventions in two case studies of infectious disease outbreaks where projections of multiple models are used to inform decision-making. Our analysis is specifically used to recapitulate the situation that many consortia find themselves in: being tasked with resolving decision-making guidance from contributions of multiple models or modelling groups. The four vote-processing rules we use are (i) First-past-the-post (FPP), (ii) Borda count, (iii) Coombs method, (iv) Alternative Vote (AV) (details of each vote-processing rule are provided in the Methods section). We believe this is the first published example of aggregating the rankings of interventions (not aggregating projections) from output of infectious disease models.

Each of the vote-processing rules investigated are single-winner voting systems that are applied using either a single list of ranked interventions (Borda Count, Coombs Method, AV), or using a list of approved interventions that pass some criteria (FPP). Each method for combining votes is outlined in the Methods below. Other vote-processing rules have been highlighted in environmental decision-making [28] and are also amenable to the approach we present here.

Several desirable properties of rank-based vote-processing rules have been discussed in the literature. We highlight four of these properties that contrast benefits and drawbacks of the vote-processing rules used in this analysis: universal domain, majority consistency, Condorcet consistency and homogeneity (table 2). ‘Universal domain’ is the property that the vote-processing rule produces a winner (or a tie) for all possible rankings that may occur [29]. We force all ‘votes’ from each model to be an ordered list (as outlined in the Methods), including a preference for each intervention available, so we do not consider a situation where the universal domain requirement is not met. ‘Majority consistency’ in a voting rule is the property that if an intervention is the preferred choice by a majority of votes, then the rule will pick the same winner under the rule in question. At face value, this seems like a reasonable property for a voting system to have. However, many scoring systems that determine a winner based upon a score that is calculated as a function of their votes do not possess this property (for example, see Borda Count, below). The property of ‘Condorcet consistency’ is determined by looking at all pairwise comparisons of interventions and then determining if any intervention would win if pitted against any other intervention (based upon majority rule) [29,30]. Such an intervention is called the ‘Condorcet winner’ but does not always exist. A vote-processing rule that also chooses the Condorcet winner is said to possess Condorcet consistency. ‘Homogeneity’ in a rank-based vote-processing rule is the property that the same winner will be determined if any number of multiples of the same sequence of votes is provided (proportions of preferences matter, not absolute numbers of preferences). Note that there is a literature on properties of vote-processing rules and that there are several other properties that we have not outlined here (e.g. [28,29]).

**Table 2.** Properties of vote-processing rules. A black circle indicates that the vote-processing rule possesses that property, no symbol indicates the vote-processing rule does not possess that property.

vote-processing rule	majority consistency	Condorcet consistency	homogeneity
First-past-the-post	•		•
Borda Count			•
Coombs Method	•	•	•
Alternative Vote	•		•

Throughout this document we use the term ‘rank’ or ‘rankings’ to refer to the numerical ordering of interventions according to some management objective (i.e. minimize outbreak duration) that is generated by comparing the projections of interventions from a single model (ranks may take non-integer values, e.g. in the case of ties). We use the term ‘vote’ to refer to the ranks after they have been processed to resolve any ties (votes can be considered as vectors of integers), and a vote is made up of ‘preferences’ (first preference, second preference, last preference, etc.). We use the term ‘winner’ to refer to the output of applying a vote-processing rule to a sample of votes (in the case studies presented, a ‘winner’ is a type of control intervention).

## 2. Methods

### (a) Case studies

We use data from two previously published analyses that use multiple models to inform decision-making. The first case study modelled a hypothetical outbreak of FMD in a county resembling Cumbria in the UK [31]. Data were generated from four spatial, stochastic, simulation models that have been used in preparedness planning of various governments: the North American Animal Disease Spread Model in the US (NAADSM), AusSpread in Australia, Interspread Plus in New Zealand and The Warwick Model in the UK. We included this case study to contrast model structures to the second case study (all compartmental models) and to highlight the impact of model stochasticity, given all of the models included in this example included stochasticity in the projections they contributed. Each model used the same starting conditions for the outbreak, the same landscape and modelled the outcomes of five interventions for controlling the outbreak, recording the final number of animals culled and the duration of the outbreak. The following interventions were simulated: culling of infected premises only (IP), culling of infected premises and dangerous contacts (IPDCs), ring culling at 3 km around infected premises (RC), ring vaccination at 3 km around infected premises (V03) and ring vaccination at 10 km around infected premises (V10). The control interventions of RC, V03 and V10 all included culling of both infected premises and culling of premises deemed dangerous contacts also. A fifth model only simulated four of the interventions. The analysis presented in this work requires all models to be able to model each intervention so that ordinal ranks are comparable across models (so-called ‘partial ballots’, where each model would vote on different subsets of interventions are mentioned later in the paper), and so this fifth was excluded from the analysis. Each model simulated each intervention 100 times. The value of 100 replicates is somewhat arbitrary in this instance; it is only used as an example of when there are multiple simulations for each model. There is no standard recommendation for the appropriate number of iterations and the number used in practice may be limited by computational resources. All models are anonymized, and data are available from the following repository: https://github.com/p-robot/objectives_matter. See Probert *et al*. [31] for further details of the simulations.

The second case study generated data from 37 stochastic compartmental models that were recapitulations of models used in the 2014 Ebola outbreak in West Africa [6]. These models were all compartmental models, but varied widely with regards to model structure and parameterization. Details of each of the models are published in Li *et al*. [6]. Models reported mean caseload from simulations in a population of 10 000 individuals using the same starting conditions. In this case study there was only a single projection for each intervention. Each model made caseload projections under five interventions: reducing community transmission by 30% (RCT; e.g. by providing sanitation kits, improved contact tracing, stronger self-isolation of sick individuals), reducing funeral transmission by 30% (RFT; e.g. by encouraging safer burial practices), reducing hospital transmission by 30% (RHT; e.g. by improving the use of personal-protective equipment by healthcare workers, and reducing hospital visits), reducing mortality ratio by 30% (RMT; e.g. reducing the case fatality ratio by improved clinical care), increasing hospitalization proportion by 30% (IHP; e.g. increasing hospital usage by increasing the number of hospital beds, or improving contact tracing) and no management (NM). See Li *et al*. [6] for further details of the models and the interventions; the data are available from the following repository: https://github.com/p-robot/voting_systems_epi_analysis.

### (b) Generating votes from model projections

In order to apply vote-processing rules to model output, votes were generated from within-model rankings of interventions. For the Ebola case study, there was one projected value per intervention from each model (the mean projected caseload for that intervention) and there were no ties in these mean values, so votes and the within-model rankings of interventions were synonymous.

For the FMD case study there are multiple simulations per model and there are ties in rankings of several of the interventions. In order to generate a list of ‘votes’ we randomly grouped output from the models into sets, where each set has the output from one of each of the interventions. As model output was generated 100 times, we obtained 100 such sets for each model. Within the results for each model, for each of these 100 replicates we then ranked each intervention according to the final number of cattle culled (minimum final number of cattle culled gets the first ranking) and according to outbreak duration (minimum outbreak duration gets the first ranking). In all cases, ties in the minimum number of cattle culled were resolved by first ranking on outbreak duration. If a preference in the best intervention was still tied, randomization was used to resolve ties. Similarly, for a primary objective of minimizing outbreak duration, ties were first resolved using a secondary objective of minimizing cattle culled, and then using randomization. Randomization has been documented as being used in case of ties in real elections [32]. Each model then contributes 100 ‘votes’ which were combined across all models using electoral vote-processing rules (so four models contributed 400 ‘votes’ overall). We note that distilling model output to ranks will ignore the predictive performance of models (a commonly used approach to weight models in an ensemble), however, vote-processing rules could still be applied to a set of votes generated in proportion to such predictive weights.

### (c) Vote-processing rules

We describe and apply four voting systems for combining recommendations across models, outlined below.

#### (i) First-past-the-post

In FPP systems, voters choose a single best intervention. The intervention with the most votes wins. This rule is also called plurality voting or simple majority.

This system of counting votes finds the majority favourite, and implementation of this system is simple and intuitive. FPP has been criticized that votes may be seen as ‘wasted’ [33,34], in the sense that if there are many possible interventions, only one will get selected and preferences for any other intervention is not included. In electoral systems, FPP has been criticized for resulting in majoritarian governments where only two large parties dominate elections and coalitions are rare. Using this system to resolve conflicts between recommendations of different interventions from models will ignore input from all models that do not have a high proportion of first preference votes; information from additional models is wasted. This method requires that models can generate a single, best intervention (in contrast to other methods that require models generate a complete ranked list of interventions). FPP does not possess the Condorcet Consistency property.

FPP is used to elect the Members of Parliament of the House of Commons of the United Kingdom [35], to elect executive and legislative representatives in the United States [27] and to pass some measures in the United States Senate [36].

#### (ii) Borda Count

Under the Borda Count method, each intervention is given points based upon voting preferences. If there are A different interventions then an intervention is given A points for each first preference vote, A − 1 points for each second preference vote and so on. Points are then tallied for each intervention and the intervention with the highest number of votes is declared a winner [29,37].

The Borda Count method is easy to implement and simple to understand. Borda Count does not possess the properties of majority consistency or Condorcet consistency, and can be influenced by the number of votes being counted. Borda Count can be thought of as a scoring system, which have been criticized before in decision-making contexts [38].

Several examples of Borda Count in environmental decision-making are given in Burgman *et al*. [28] and a variation of Borda Count is used in the Eurovision song contest to select a winner [39].

#### (iii) Coombs Method

Under the Coombs Method, first preference votes are counted. If an intervention has an absolute majority (greater than 50%) of first preferences, then that intervention is declared the winner. If no winner is declared then the intervention with the highest proportion of last preference votes is eliminated [29]. The algorithm for the Coombs Method then continues with multiple rounds of counting and elimination (as above) until a winner is found. The Coombs Method possesses all the properties of a vote-processing rule that we outline.

Alternative versions of the Coombs Method have been proposed that do not include a check of whether a majority is found and start by eliminating interventions [29]. However, such modifications to this method do not possess majority consistency and we do not consider them further. We are not aware of real-world applications of the Coombs Method but include it to contrast the other rules as it possesses all the properties in table 2.

#### (iv) Alternative Vote

Using the AV method each vote is a ranked list of interventions. All first preferences are tallied; if an absolute majority of voters (greater than 50%) choose a single intervention as their first preference then counting stops and this intervention is chosen as the winner. If no winner is found, then the intervention with the smallest proportion of first preferences is eliminated. For each vote that ranked the eliminated intervention as first preference, these first preference votes are transferred to the intervention that was ranked as the next highest preference [29,33]. The algorithm continues with rounds of counting and elimination until a winner is found. This method can also be described as the single transferable vote (STV) when only choosing one option [29,32]. It is also called Instant Run-off Voting (in the US) or Preferential Voting (in Australia) [40]. The Coombs Method differs from the AV in how interventions are eliminated in each round: AV eliminates interventions with the least proportion of first preference votes, the Coombs Method eliminates interventions with the highest proportion of worst preference votes [41].

The AV method has been noted as being susceptible to tactical voting by eliminating a candidate via secondary preferences [40]. The AV method does not possess the property of Condorcet consistency.

The AV method has been used to elect the Australian House of Representatives since 1918, in Irish Presidential elections [42], and to elect chairs of select committees in the House of Commons in the UK [35].

### (d) Sensitivity analyses

Analyses of multiple models can be complicated in a number of ways, including sensitivity to the addition of new models. To test the robustness of using voting systems to select actions from model sets, we investigated the effect of adding two different types of pathological models to the final ballot of votes to see how this would affect the choice of a winner.

First, we investigated adding models that randomly allocate preferences to each intervention. This approach represents the extreme case of adding non-informative models, i.e. models that have no predictive ability, and aims to address concerns that adding such models may sabotage multi-model analyses. For each case study, we added *N* models with randomly allocated preferences (and for each objective within the FMD case study), where *N* ranged from 1 to *M*, and *M* was 10 times the size of the original cohort of models (*M* = 50 in the FMD case study, *M* = 370 in the EV case study). For a fixed number of added models, *N*, we repeated the experiment 100 times and report the proportion of times that each intervention is chosen as the winner using each vote-processing rule. In the FMD case study we added votes in multiples of 100 (as per the contributions of each original model) whereas in the Ebola case study we added single votes.

Second, we investigated the impact of adding ‘biased’ models to the original cohort of models in each case study. We defined a ‘biased’ model as that which favoured a single intervention for every vote (i.e. gave 100% of first preferences to a single model). This situation may arise in the context of mathematical modelling in a number of ways, for instance when several models have a similar structure (e.g. the cohort of models is dominated by compartmental models) and thereby could all favour a single intervention. This is similar to investigating the susceptibility of a rule to tactical voting. Beyond the biased intervention, these models would allocate preferences towards the other interventions randomly. For each case study, we added *B* ‘biased’ models, where *B* ranged from 1 to *C*, where *C* was the size of the original cohort of models (*C* = 4 and *C* = 37 in the FMD and Ebola case studies, respectively). For a fixed number of added ‘biased’ models, *N*, we repeated the experiment 100 times and report the proportion of times that each intervention is chosen as the winner using each vote-processing rule. We repeated this experiment for each candidate—that is, adding models that favoured each of the candidate interventions in turn.

The inclusion of the two sensitivity analyses highlights the impact that adding additional models can have on the overall winner, and from which it may be possible to comment on the impact of model stochasticity on these vote-processing rules: at one extreme, highly stochastic models that randomly choose the best intervention, and at the other extreme models with very small stochasticity that always choose the same best action.

Python code for generating ‘votes’ from model output, and for implementing each of the voting systems, is documented in a Python module available at https://github.com/p-robot/voting_systems. The complete analysis in this manuscript, which uses the voting_systems module, is available at: https://github.com/p-robot/voting_systems_epi_analysis. The voting_systems Python package is tested using the pytest package, includes 71 tests, and the complete analysis requires Python greater than 3.6. The 71 tests in the package run each vote-processing rule through 10–20 different example ballots, comparing expected outputs with those generated by the code. The github repository includes a slide deck illustrating the algorithms of the four vote-processing rules and an illustration of a subset of the tests used to validate the coded algorithms of the vote-processing rules.

## 3. Results

In the FMD case study, all vote-processing rules returned the same winner for an objective of minimizing total cattle culled—ring vaccination at 10 km. FPP (majority rule) chose ring vaccination at 10 km as the best control intervention since 42.1% of votes chose this as their first preference (table 3). Borda Count gave highest points to ring vaccination at 10 km (1635 points), followed by vaccination at 3 km (1527 points), IP culling (1200 points), IPDC (935 points) and finally ring culling at 3 km (688 points). The AV and the Coombs Method both eliminated the same series of interventions until ring vaccination at 10 km had a majority. Table 3 summarizes the proportion of votes in which each intervention had a particular ranking and therefore also highlights the impact of stochasticity in model output (all models in the FMD case study are stochastic).

**Table 3.** Percentage of votes of each preference (columns) for each control intervention (rows) from 100 simulations from four models in a hypothetical FMD outbreak in a county in the UK. Two objectives are presented for ranking model projections: minimizing total cattle culled and minimizing outbreak duration. Control interventions are infected premises culling (IP), infected premises culling and dangerous contacts culling (IPDC), ring culling at 3 km around infected premises (R3), ring vaccination at 3 km (V3) and 10 km (V10) around infected premises. For instance, 16.3% of votes had IP culling as their first preference for minimizing cattle culled.

objective	intervention	1st	2nd	3rd	4th	5th
minimize total cattle culled (head)	IP	16.3	19.5	31.0	14.8	18.3
	IPDC	7.5	8.3	16.8	45.9	21.6
	RC3	3.8	2.8	12.0	25.1	56.4
	V3	30.3	36.3	21.6	9.3	2.5
	V10	42.1	33.1	18.5	5.0	1.3
minimize outbreak duration (days)	IP	7.0	17.5	22.3	22.3	30.8
	IPDC	11.5	19.8	19.5	24.6	24.6
	RC3	55.9	8.5	7.0	12.3	16.3
	V3	11.3	24.1	27.1	23.1	14.5
	V10	14.2	30.1	24.1	17.8	13.8

Under an objective of minimizing outbreak duration, all vote-processing rules also returned the same winner for an objective of minimizing outbreak duration—ring culling at 3 km. FPP (majority rule) chose ring culling at 3 km as the winning intervention since 55.9% of votes chose this intervention as their first preference (table 3). Borda Count gave highest points to ring culling at 3 km (1498 points), followed by vaccination at 10 km (1250 points), vaccination at 3 km (1175 points), IPDC (1074 points) and finally IP culling (988 points). The AV and the Coombs Method did not eliminate any candidate interventions as an absolute majority (55.9% of first preferences for ring culling at 3 km) was met in the first round.

Ranking interventions based upon mean projections of output across all models produced the same results as using vote-processing rules (electronic supplementary material, table S1).

In the Ebola virus case study, all vote-processing rules returned the same winner for an objective of minimizing caseload—RFT by 30%. FPP (majority rule) chose this as the winner since 59.5% of models chose this as their first preference (table 4). Borda Count gave highest points to RFT by 30% (197 points), followed by RCT by 30% (191 points), reducing the mortality ratio by 30% (158 points), RHT by 30% (93 points), IHP by 30% (91 points) and finally RCT by 30% (47 points). The AV and the Coombs Method did not eliminate any candidate interventions as an absolute majority (59.5% of first preferences for RFT) was met in the first round.

**Table 4.** Percentage of votes of each preference (columns) for each control intervention (rows) from 37 models of the 2014 Ebola outbreak in 2014. Simulations were of caseload projections in a simulated population of 10 000 individuals. Control interventions are reducing community transmission by 30% (RCT), reducing funeral transmission by 30% (RFT), reducing hospital transmission by 30% (RHT), reducing mortality ration by 30% (RMT), increasing hospitalization proportion by 30% (IHP) and no management (NM). For instance, 91.9% of votes had RCT as the first or second preference intervention.

objective	intervention	1st	2nd	3rd	4th	5th	6th
minimize caseload	RCT	27.0	64.9	5.4	2.7	0.0	0.0
	RFT	59.5	16.2	21.6	2.7	0.0	0.0
	RHT	5.4	0.0	2.7	27.0	62.2	2.7
	RMR	8.1	18.9	64.1	2.7	2.7	0.0
	IHP	0.0	0.0	2.7	62.2	13.5	21.6
	NM	0.0	0.0	0.0	2.7	21.6	75.7

In the Ebola virus case study, ranking interventions based upon mean projections of output across all models ranked reduction in community transmission (RCT) as the best intervention, in contrast to using vote-processing rules (electronic supplementary material, table S2).

Figures of the raw model projections in each case study are provided in electronic supplementary material, figures S1 and S2.

### (a) Ties

In generating votes from model projections, ties may occur. Ties in rankings occurred in 20% of total votes in the FMD case study when ranking on projections of total cattle culled. This was largely driven by the results from one model (most models had less than 5% of rankings causing ties). Of those that needed to be resolved via a secondary objective (outbreak duration), 32.93% were still tied (6.77% overall) and had to have ties resolved via randomization. That is, for 6.77% of the 100 model replicates that were used to form votes, there was no clear preference in the best control intervention for minimizing total cattle culled even after a secondary ranking on outbreak duration. There were no ties in the rankings of projections of caseload in the 37 Ebola models.

### (b) Sensitivity of voting rules to addition of successive models that rank interventions randomly

**Figure 2.**
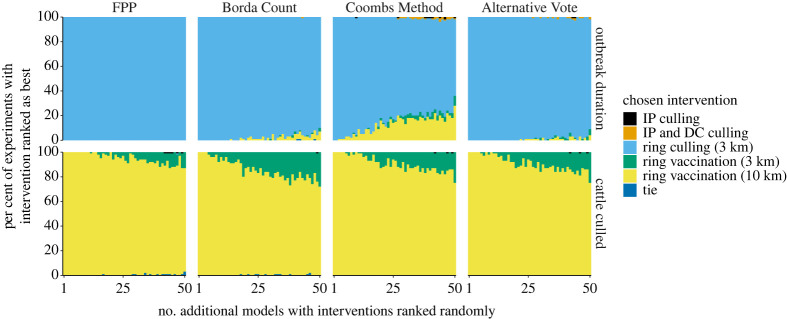
Sensitivity of recommended control intervention under different vote-processing rules when successive models with randomly allocated rankings of interventions are included in an ensemble, FMD case study. Within each panel, the horizontal axis is the number of additional models with randomly allocated rankings that have been added, the vertical axis shows the proportion of times, within 100 simulated experiments, that each control intervention was recommended as the best. Rankings are combined from five interventions from a number of model (four models plus a number of additional models that rank intervention randomly, as shown on the horizontal axis) of FMD and using four vote-processing rules (panel columns). Output is shown across three different control objectives (panel rows; that is, the metric upon which control interventions were ranked). Interventions have been stacked in a consistent manner (as shown in the order in the legend). (Online version in colour.)

Adding models that ranked interventions in a random order made no difference to the chosen winning intervention under all voting rules until differences caused by stochasticity were larger than the original absolute difference in tallies of preferences (figure 2). This threshold depended upon the vote-processing rule, the metric upon which interventions were being ranked (the objective) and the tally of votes for the additional interventions in the original cohort of models (i.e. those tallies in tables 3 and 4). In the FMD example, Borda Count and the Coombs Method seemed most susceptible to the effect of adding additional models with randomly allocated rankings. Under these two rules it took the smallest number of models added until the recommended intervention was changed and both rules resulted in the largest proportion of adjustments when a large number of models were added. FPP was least susceptible to the addition of random models, due to the large differences in first and second preferences in the original cohort of votes (table 3). The Coombs Method and the AV gave almost identical results under an objective of minimizing cattle culled but quite different patterns when ranking interventions on outbreak duration. This is due to the distribution of first and last preferences in the data (since these two rules differ in whether candidates are removed on ‘least number of first preferences' or ‘largest number of worst preferences’). That is, in table 3 the proportion of first and last preferences under cattle culled are perfectly inversely correlated (first preferences are ordered RC < IPDC < IP < V3 < V10 whereas last preferences are ordered RC > IPDC > IP > V3 > V10) but under an objective of outbreak duration, the orderings of the first and last preferences are not so aligned. Overall, all vote-processing rules were reasonably robust to the addition of random models—even after 10 times the number of models in the original cohort were added in the FMD case study, the same control intervention was chosen over 60% of the time under all voting rules as was chosen when no ‘random’ models were added.

**Figure 3.**
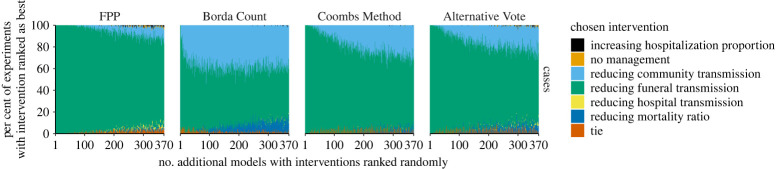
Sensitivity of recommended control intervention under different vote-processing rules when success models with randomly allocated rankings of interventions are included in an ensemble, Ebola case study. Within each panel, the horizontal axis is the number of additional models with randomly allocated rankings that have been added, the vertical axis shows the proportion of times, within 100 simulated experiments, that each control intervention was recommended as the best. Rankings are combined from six interventions from 37 models of Ebola and using four vote-processing rules (panel columns). Interventions have been stacked in a consistent manner (as shown in the order in the legend). (Online version in colour.)

Within the Ebola case study, FPP was most resilient to the addition of randomly ranked models (figure 3). Borda Count was highly susceptible to the addition of extra models with ‘RCT by 30%’ as the second most common choice of a winner due to the large proportion of 2nd preference votes for this intervention and therefore a large Borda Count score (table 3). The Coombs Method and AV provided similar results.

### (c) Sensitivity of voting rules to addition of successive models biasing a single model

**Figure 4.**
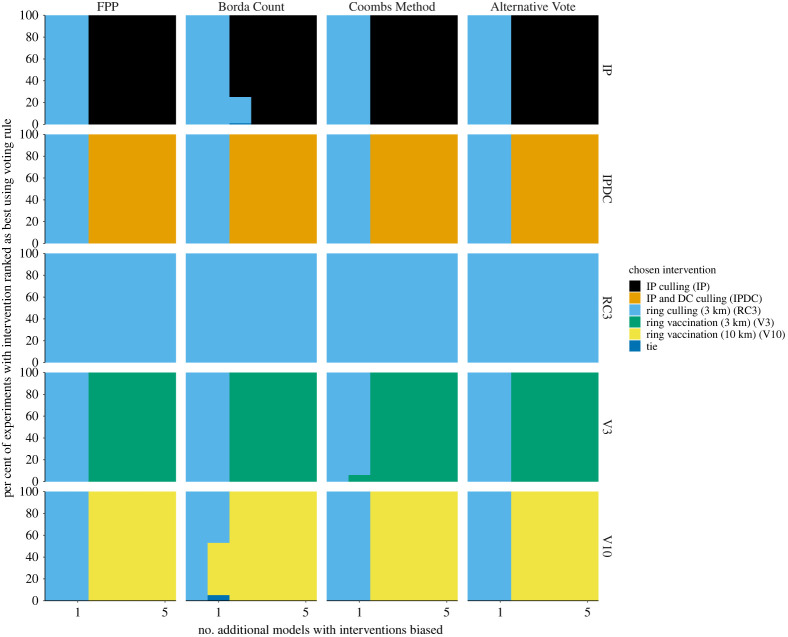
Chosen recommended intervention under a range of vote-processing rules when additional biased models are included in a multi-model analysis of FMD. Results shown for an objective of minimizing outbreak duration. Biased models are defined as those which favour only one intervention as best and randomly rank the other interventions. Within each panel, the horizontal axis is the number of biased models added, the vertical axis is the per cent of time a chosen intervention is chosen as the winner under a particular vote-processing rule (columns). Rows denote the intervention that was biased (indexed as in the legend). The first and second most preferred models in the underlying data are ring culling (3 km) and ring vaccination (10 km) (table 3). (Online version in colour.)

**Figure 5.**
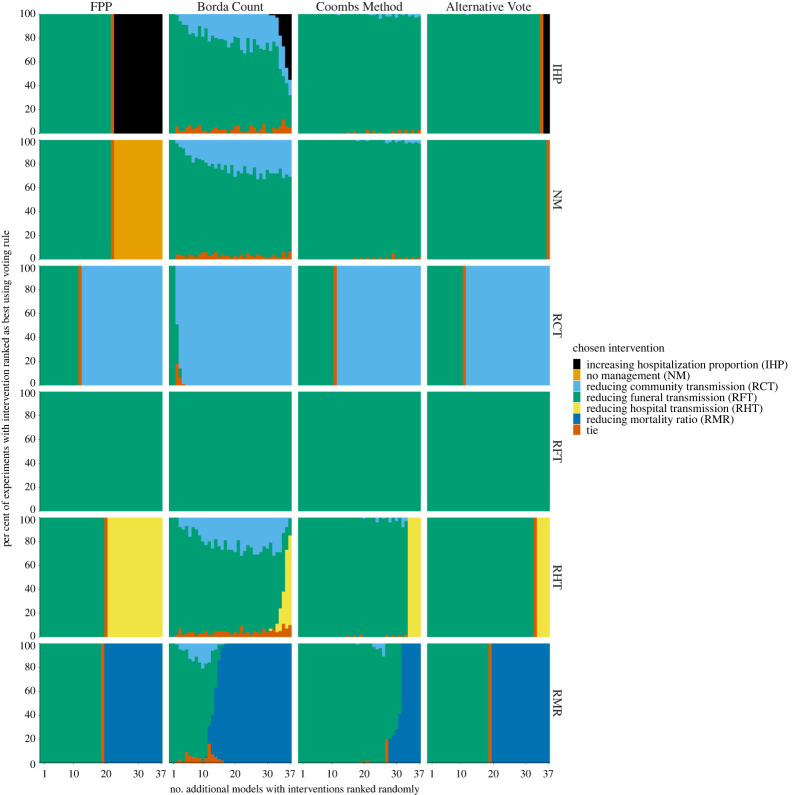
Chosen recommended intervention under a range of vote-processing rules when additional biased models are included in a multi-model analysis of Ebola. Biased models are defined as those which favour only one intervention as best and randomly rank the other interventions. Within each panel, the horizontal axis is the number of biased models added, the vertical axis is the per cent of time a chosen intervention is chosen as the winner under a particular vote-processing rule (columns). Rows denote the intervention that was biased (indexed as in the legend). (Online version in colour.)

For both case studies, the number of biased models that were needed to add to change the winner (figures 4 and 5, electronic supplementary material, figure S3) largely reflected the distribution of first preferences votes in the original cohort (tables 3 and 4). That is, in both case studies, biased models only mattered in situations where they were favouring an intervention that already had reasonable support (such as the second most favoured intervention). In the FMD case study, for an objective of minimizing outbreak duration, one to two biased models were required to change the winner for all voting methods (figure 4). For an objective of minimizing cattle culled, the intervention with the second largest proportion of first preference votes is ring vaccination at 3 km (30.3%; table 3) and it was the intervention that was chosen as the winner most quickly (i.e. with the smallest number of additional models added) (electronic supplementary material, figure S3).

In the Ebola case study, Borda Count was highly susceptible to the addition of biased models to the original cohort of models (figure 5). Borda Count adjusted the winner earlier than the other vote-processing rules and included more uncertainty in its choice of winning intervention. This highlights how the allocation of second and third preferences within votes has a large impact when using the Borda Count method.

## 4. Discussion

We present the application of electoral vote-processing rules for combining rankings of control interventions when multiple epidemiological models are used to make projections of different interventions. Combining rankings of interventions from each model may be desirable compared to directly comparing projections from different models for a number of reasons. Firstly, no implicit assumptions are made regarding the comparability of projections across models. Directly comparing or averaging model outputs based upon common language terms, such as ‘hospitalized cases’, belies the potentially vastly different calculations and assumptions that lead to these separate outputs. Such differences are likely to be more prevalent as model complexity increases and the variability in model complexity increases within ensembles of models. Secondly, we allowed models to ‘vote’ on the performance of control interventions under different control objectives, thereby keeping hidden assumptions constant across such projections. Thirdly, by not combining projections across models we avoid models with very large (or small) projections from undue influence on the aggregate (e.g. mean) projection. We applied the approach in two case studies, one in human and one in animal health, that differed in how this method was applied: one allowed each model to ‘vote’ multiple times (via stochastic model replicates), the other allowed models to vote once (using the mean projected value). The two illustrated approaches are not an exhaustive list of ways in which vote-processing rules can be applied to model output but are illustrative of the issues and benefits of this general approach.

In each case study, each of the four vote-processing rules we investigated selected the same preferred control intervention. FPP (or ‘plurality voting’) was most resilient to the process of adding additional models with randomly ranked interventions. Borda Count, a scoring rule, was generally most susceptible to adding random models, with susceptibility to changing winner depending particularly on the second preferences (the unsuitability of scoring rules in a decision-making context has been highlighted elsewhere; e.g. [38]). The Coombs Method and AV had similar patterns when adding random models but, again, the outcome depended on the distribution of first and last preference votes (as this is how these rules decide which candidates to remove). Including biased models that favoured a single intervention only mattered in our analysis when the second most favourite intervention was the biased intervention. Borda Count, a scoring rule, was the most susceptible to the addition of biased models in our case studies. Although the four vote-processing rules selected the same control intervention as the winner in the two case studies we investigated, we do not believe this is a universal property of applying vote-processing rules to output from epidemiological models, and believe this to be specific to the presented case studies.

Ranking based upon overall mean projection of interventions produced the same results as the vote-processing rules for the FMD case study but a different ranking in the Ebola virus case study, where the intervention of ‘RCT’ had the lowest mean projected caseload. This result underscores the utility of preserving comparisons in control interventions to only be within-model comparisons by combining rankings of interventions generated by models. Using rankings in this manner avoids model-averaged projections being unduly influenced by outliers, and allows the second (and later) preferences of models for particular control interventions to have an influence on the final recommendation.

The presented analysis shed light on how model stochasticity may affect outcomes of using vote-processing rules. Model stochasticity is partially taken into account in two ways: (i) by inclusion of the FMD case example, and (ii) by including the sensitivity analyses. First, we included the FMD example to highlight the impact of model stochasticity, given all of the models included in that example are stochastic and therefore included stochasticity in the projections they contributed (table 3 summarizes the proportion of votes in which each intervention had a particular ranking and therefore highlights the impact of this stochasticity). Second, the inclusion of two sensitivity analyses highlights the impact that adding additional models can have on the overall winner, from which one may deduce the impact of stochasticity on these vote-processing rules—at one extreme, highly stochastic models that randomly choose the best intervention and at the other extreme models with very small stochasticity that always choose the same best action. The impact of parameter uncertainty is much harder to gauge in our analysis since each case study includes numerous models and each model has a different number of parameters.

Considering voting rules for evaluating epidemic interventions across multiple models suggests several exciting avenues for future research. As yet, we can offer no guidance on how many models may be needed to inform decision-making. Vote-processing rules will become unpredictable with very few models (in which case we have illustrated the use of model replicates with stochastic models) to generate votes, but what is the minimum for stable results? We have implemented four vote-processing rules that are simple to compute and commonly used but many other vote-processing rules exist and are used in decision-making (e.g. [28]); a thorough exploration would provide guidance on what approaches are best used in specific situations. It may also be valuable to assess the best approach when we have a blend of deterministic and stochastic models, or when we may have both stochastic and parametric uncertainty, as is common in wide-ranging multi-model elicitations. Although we do not provide criteria for which models to include (that is not the topic of this research, see [1,20]) and that models may have very different assumptions, we believe our study provides some evidence for including a range of models when making aggregate predictions. This is in line with other studies in the expert elicitation literature that showed using broadly defined expert groups led to better calibrated results [22].

We highlighted several relevant properties of the vote-processing rules when applied to output from epidemiological models but several additional properties may be of interest [29]. Lucidity, or how straightforward the rule is to understand, is important in communicating results to policymakers and the public. The ease of computing what constitutes a ‘vote’ is also important in communicating results to stakeholders in the decision-making process. We only considered two methods of generating votes from model output: (i) where a ‘vote’ is a ranking of control interventions and (ii) where a ‘vote’ is a single preferred control intervention. Both methods for generating votes could be modified in different ways, such as using different functions for determining a single preferred control intervention (e.g. [31]). The computational overhead in generating votes, and also in processing votes, is particularly relevant if these techniques are to be used for real-time decision-making. However, the computational overhead of the vote-processing rules that we present is negligible compared with the computational overhead of running each individual model. Finally, resistance to tactical voting is another desirable property of vote-processing rules [29]. Tactical voting, when ranking model outcomes, may occur deliberately or accidentally, for example, through the addition of new models that are insufficiently different from models in the existing set, and it is, therefore, worth considering the situations in which a vote-processing rule may be susceptible to tactical voting (e.g. see discussion surrounding Borda Count in Methods). Our investigation of biased and random models suggests that substantial interference is required to change the best decision in our case studies, but in examples where the difference between the first- and second-ranked actions is close, additional scrutiny should be applied to avoid biases in the model set influencing the result. Leave-one-out cross validation (i.e. computing the best decision in the absence of each model in the ensemble) is a simple test to confirm the influence of a single model on the results and would be a good check to perform in the case where model bias is suspected.

We considered situations where each model contributed the same number of ‘votes’ and that ‘votes’ are generated from model replicates. There are many ways in which votes could be generated from model output. If the models are related or if there is reason to weight one model differently from other models, these methods could still be applied by generating a number of replicates in proportion to each model's ‘weight’ (for instance a weight according to their predictive accuracy).

Further, we have only illustrated a decision-making process where choice of intervention neatly falls into a discrete set of choices. This is a simplification of real decision-making for the purposes of illustrating a technique. There are clearly decisions that do not easily fall into different categories as we have investigated here. In both case studies, the distribution of first preferences favoured a single model in the original cohort of models. The impact of using different vote-processing rules will become more apparent when the differences between interventions is more nuanced.

The techniques we present did not deal with partial or truncated ballots [43,44]. We investigated sensitivity analyses when models consistently ‘bias’ a single first preference but did not investigate when models may consistently ‘bias’ a single worst preference. Voting methods exist for the situation where some models may not be able to evaluate all control interventions (e.g. [6,31]), so-called truncated polling. The dominant methods for assigning votes to truncated ballots assume that all candidates (or models) were available to the voter, but that the voter simply did not express votes for some candidates [45]. This is not the case when models cannot evaluate all control interventions, which is more analogous to some voters having only a restricted set of candidates to choose from (e.g. partial ballots). To our knowledge, the voting literature does not consider this situation and tools for evaluating winners in this situation do not exist. A useful extension to this work would be to develop methods for assigning votes to model sets that consider subsets of control interventions.

We have only considered the situation where a single winner was to be chosen. However, methods also exist for choosing multiple interventions at once, such as the STV method.

One of the arguments for application of voting methods is to avoid direct comparison of model projections. However, combining ranks of interventions across models will compare control interventions and these are also subject to interpretation (e.g. linguistic uncertainty in control interventions in [1,21]) and so this does not detract from the need for communication to modellers to be very specific with regards to the details of the control interventions being modelled.

In summary, by comparing ranking of interventions instead of directly comparing projections of models we avoid potentially combining metrics that are not representing the same epidemiological measure. Comparing rankings means that all comparisons of absolute projections are kept as within-model comparisons. The methods presented here provide another fast and straightforward avenue for comparing the output from epidemiological modelling exercises, particularly in the case where there are multiple models informing a decision-making process. Voting methods represent another useful approach in the modeller's toolbox to be used in concert with other established ensemble modelling approaches. Given that multi-model efforts are becoming increasingly common [1,3,4,5,7,21], best methods to collate and interpret their outputs for decision-makers is essential.

## Data Availability

All data are either already published (the two case studies) or are generated completely by the code provided. The manuscript Methods section details the code and it is hosted on a publicly available repository. The data are provided in the electronic supplementary material [46].

## References

[1] Shea K *et al.* 2020 COVID-19 reopening strategies at the county level in the face of uncertainty: Multiple Models for Outbreak Decision Support. Medrxiv. (10.1101/2020.11.03.20225409)

[2] Reich NG *et al.* 2019 Accuracy of real-time multi-model ensemble forecasts for seasonal influenza in the U.S. PLoS Comput. Biol. **15**, e1007486. (10.1371/journal.pcbi.1007486) 31756193 PMC6897420

[3] Kao RR. 2002 The role of mathematical modelling in the control of the 2001 FMD epidemic in the UK. Trends Microbiol. **10**, 279-286. (10.1016/S0966-842X(02)02371-5) 12088664

[4] Pomeroy *et al.* 2015 Data-driven models of foot-and-mouth disease dynamics: a review. *Transbound. Emerg. Dis*. **64**, 716-728. (10.1111/tbed.12437) PMC5205574 26576514

[5] Eaton *et al.* 2015. Assessment of epidemic projections using recent HIV survey data in South Africa: a validation analysis of ten mathematical models of HIV epidemiology in the antiretroviral therapy era. Lancet Glob. Health **3**, e598-e608. (10.1016/S2214-109X(15)00080-7) 26385301

[6] Li SL, Bjørnstad ON, Ferrari MJ, Mummah R, Runge MC, Fonnesbeck CJ, Tildesley MJ, Probert WJ, Shea K. 2017 Uncertainty and optimal control of Ebola outbreaks. Proc. Natl Acad. Sci. USA **114**, 5659-5664. (10.1073/pnas.1617482114) 28507121 PMC5465899

[7] Borchering RK *et al.* 2021 Modeling of future COVID-19 cases, hospitalizations, and deaths, by vaccination rates and nonpharmaceutical intervention scenarios - United States, April–September 2021. MMWR Morb. Mortal. Wkly. Rep. **70**, 719-724. (10.15585/mmwr.mm7019e3externalicon) 33988185 PMC8118153

[8] Cramer EY *et al.* 2021 The United States COVID-19 Forecast Hub dataset. medRxiv. (10.1101/2021.11.04.21265886) PMC9342845 35915104

[9] SPI-M-O: Medium-term projections and model descriptions. 2020 Supporting document prepared by the Scientific Pandemic Influenza Group on Modelling, Operational sub-group. Date published: 27 November 2020. Date accessed: 30 August 2021. See https://www.gov.uk/government/publications/spi-m-o-covid-19-medium-term-projections-explainer-31-october-2020.

[10] Li X *et al.* 2021 Estimating the health impact of vaccination against ten pathogens in 98 low-income and middle-income countries from 2000 to 2030: a modelling study. Lancet **397**, 398-408. (10.1016/S0140-6736(20)32657-X) 33516338 PMC7846814

[11] Taylor KE, Stouffer RJ, Meehl GA. 2012 An overview of CMIP5 and the experiment design. Bull. Am. Meteorol. Soc. **93**, 485-498. (10.1175/BAMS-D-11-00094.1)

[12] Bates JM, Granger CWJ. 1969 The combination of forecasts. J. Oper. Res. Soc. **20**, 451-468. (10.1057/jors.1969.103)

[13] Araújo M, New M. 2007 Ensemble forecasting of species distributions. Trends Ecol. Evol. **22**, 42-47. (10.1016/j.tree.2006.09.010) 17011070

[14] Krishnamurti TN, Kishtawal CM, LaRow TE, Bachiochi DR, Zhang Z, Williford CE, Gadgil S, Surendran S. 1999 Improved weather and seasonal climate forecasts from multimodel superensemble. Science **285**, 1548-1550. (10.1126/science.285.5433.1548) 10477515

[15] Parker WS. 2010 Predicting weather and climate: uncertainty, ensembles and probability. Stud. Hist. Philos. Sci. B: Stud. Hist. Philos. Mod. Phys. **41**, 263-272. (10.1016/j.shpsb.2010.07.006)

[16] Grenouillet *et al.* 2011 Ensemble modelling of species distribution: the effects of geographical and environmental ranges. Ecography **34**, 9-17. (10.1111/j.1600-0587.2010.06152)

[17] Anderson SC *et al.* 2017 Improving estimates of population status and trend with superensemble models. *Fish Fish*. **18**, 732– 741. (10.1111/faf.12200)

[18] Lindström T, Tildesley M, Webb C. 2015 A Bayesian ensemble approach for epidemiological projections. PLoS Comput. Biol. **11**, e1004187. (10.1371/journal.pcbi.1004187) 25927892 PMC4415763

[19] Webb C *et al.* 2017 Ensemble modelling and structured decision-making to support Emergency Disease Management. Prev. Vet. Med. **138**, 124-133. (10.1016/j.prevetmed.2017.01.003) 28237227

[20] den Boon S *et al.* 2019 Guidelines for multi-model comparisons of the impact of infectious disease interventions. BMC Med. **17**, 163. (10.1186/s12916-019-1403-9) 31422772 PMC6699075

[21] Shea K, Runge MC, Pannell D, Probert WJ, Li SL, Tildesley M, Ferrari M. 2020b Harnessing multiple models for outbreak management. Science **368**, 577-579. (10.1126/science.abb9934) 32381703

[22] Burgman MA, McBride M, Ashton R, Speirs-Bridge A, Flander L, Wintle B, Fidler F, Rumpff L, Twardy C. 2011 Expert status and performance. PLoS ONE **6**, e22998. (10.1371/journal.pone.0022998) 21829574 PMC3146531

[23] Probert WJM *et al.* 2018 Real-time decision-making during emergency disease outbreaks. PLoS Comput. Biol. **14**, e1006202. (10.1371/journal.pcbi.1006202) 30040815 PMC6075790

[24] Clemen R. 1989 Combining forecasts: a review and annotated bibliography. Int. J. Forecast. **5**, 559-583. (10.1016/0169-2070(89)90012-5)

[25] Shea K, Tildesley MJ, Runge MC, Fonnesbeck CJ, Ferrari MJ. 2014 Adaptive management and the value of information: learning via intervention in epidemiology. PLoS Biol. **12**, e1001970. (10.1371/journal.pbio.1001970) 25333371 PMC4204804

[26] Morgan MG, Henrion M. 2010 Uncertainty: a guide to dealing with uncertainty in quantitative risk and policy analysis, Revised edn. Cambridge, UK: Cambridge University Press.

[27] Amy DJ. 2000 Behind the ballot box: a citizen's guide to voting systems. Santa Barbara, CA: Praeger.

[28] Burgman MA, Regan HM, Maguire LA, Colyvan M, Justus J, Martin TG, Rothley K. 2014 Voting systems for environmental decisions. Conserv. Biol. **28**, 322-332. (10.1111/cobi.12209) 24423154 PMC4265892

[29] Tideman T. 2006 Collective decisions and voting: the potential for public choice. Aldershot, UK: Ashgate Publishing Ltd.

[30] Condorcet 1785. Essai sur l’application de l’analyse à la Probabilité des Décisions Rendues à la Pluralité des Voix. Cambridge, UK: Cambridge University Press.

[31] Probert WJ *et al.* 2016 Decision-making for foot-and-mouth disease control: objectives matter. Epidemics **15**, 10-19. (10.1016/j.epidem.2015.11.002) 27266845 PMC4902768

[32] NZ DIA (New Zealand Government Department of Internal Affairs) 2022 Single Transferable Vote website. https://www.stv.govt.nz/onevacancy.shtml (accessed 4th July 2022).

[33] Reeve A, Ware A. 1992 Electoral systems: a comparative and theoretical introduction. London, UK: Routledge.

[34] UK Electoral Reform 2018 First-past-the-post. See https://www.electoral-reform.org.uk/voting-systems/types-of-voting-system/first-past-the-post (accessed 26 October 2018).

[35] UK Parliament 2018 Voting systems in the UK. See https://www.parliament.uk/about/how/elections-and-voting/voting-systems/ (accessed 7 October 2018).

[36] US Senate 2018 Yea or Nay? See https://www.senate.gov/general/Features/votes.htm (accessed 26 October 2018).

[37] Electoral Reform Society 2018 Borda Count. Date accessed: 14 November 2018. See https://www.electoral-reform.org.uk/voting-systems/types-of-voting-system/borda-count/.

[38] Game E, Kareiva P, Possingham H. 2013 Six common mistakes in conservation priority setting. Conserv. Biol. **27**, 480-485. (10.1111/cobi.12051) 23565990 PMC3732384

[39] Eurovision 2018 Date Accessed 4 October 2018. See https://eurovision.tv/about/voting.

[40] Rose R. 2000 International encyclopedia of elections. Washington, WA: DC: CQ Press.

[41] Abramson PR, Aldrich JH, Diskin A, Houck AM, Levine R, Scotto TJ. 2013 The British general election of 2010 under different voting rules. Elect. Stud. **32**, 134-139. (10.1016/j.electstud.2012.10.002)

[42] Farrell D. 2011 Electoral systems: a comparative introduction, 2nd edn. New York, NY: Palgrave Macmillan.

[43] Kilgour DM, Grégoire JC, Foley AM. 2020 The prevalence and consequences of ballot truncation in ranked-choice elections. Public Choice **184**, 197-218. (10.1007/s11127-019-00723-2)

[44] Konczak K, Lang J. 2005 Voting procedures with incomplete preferences. In *Proc. of the Multidisciplinary IJCAI-05 Workshop on Advances in Preference Handling (M-PREF*), Edinburgh, UK, 31 July - 1 August 2005. Edinburgh, UK.

[45] Baumeister D, Faliszewski P, Lang J, Rothe J. 2012 Campaigns for lazy voters: truncated ballots. In *Proc. of the 11th Int. Conf. on Autonomous Agents and Multiagent Systems - Volume 2*, Valencia, *Spain, 4–8 June 2012*, pp. 577-584. Liverpool, UK: IFAAMAS.

[46] Probert WJM, Nicol S, Ferrari MJ, Li S-L, Shea K, Tildesley MJ, Runge MC. 2022 Data from: Vote-processing rules for combining control recommendations from multiple models Figshare. (10.6084/m9.figshare.c.6070432) PMC9376708 35965457

